# Efficient Conversion of 5-Hydroxymethylfurfural to 2,5-Furandicarboxylic Acid by the Magnetic Laccase Nanoflowers-2,2,6,6-Tetramethylpiperidin-1-Oxyl System

**DOI:** 10.3390/ma18163780

**Published:** 2025-08-12

**Authors:** Lei Yang, Anbang Duan, Zhanyin Liu, Tingying Wei, Chunzhao Liu

**Affiliations:** State Key Laboratory of Bio-Fibers and Eco-Textiles, Institute of Biochemical Engineering, College of Materials Science and Engineering, Qingdao University, Qingdao 266071, China; 15890932657@163.com (L.Y.); wingsblinkfans@163.com (A.D.); 17860100516@163.com (Z.L.); weimoer04@163.com (T.W.)

**Keywords:** laccase immobilization, magnetic hybrid nanoflowers, 5-hydroxymethylfurfural, 2,5-furandicarboxylic acid, biocatalysis

## Abstract

Aiming to address the key challenges of poor enzyme stability, difficult recovery, and difficult synergistic optimization of catalytic efficiency in high-value conversion of biomass, this study utilizes mineralization self-assembly technology to combine laccase with Fe_3_O_4_@SiO_2_-PMIDA-Cu^2+^ composite, constructing magnetic laccase nanoflower (MLac-NFs) materials with a porous structure and superparamagnetism. This synthetic material can efficiently catalyze the selective oxidation of 5-hydroxymethylfurfural (HMF) to 2,5-furandicarboxylic acid (FDCA). The characterization results indicated that MLac-NFs exhibit optimal catalytic activity (63.4 U mg^−1^) under conditions of pH 6.0 and 40 °C, with significantly enhanced storage stability (retaining 94.26% of activity after 30 days of storage at 4 °C). Apparent kinetic analysis reveals that the substrate affinity and maximum reaction rate of MLac-NFs were increased by 38.3% and 439.6%, respectively. In the laccase–mediator system (LMS), MLac-NFs mediated by 30 mM TEMPO could achieve complete conversion of HMF to FDCA within 24 h. Moreover, due to the introduction of magnetic nanoparticles, the MLac-NFs could be recovered and reused via an external magnetic field, maintaining 53.26% of the initial FDCA yield after six cycles.

## 1. Introduction

The development of innovative, economical, and efficient green catalytic processes to reduce environmental loads and industrial costs is a core goal in the field of sustainable chemistry. Among the biomass platforms represented by lactic acid and glucose, 5-hydroxymethylfurfural (HMF) is considered a potential bio-based platform molecule due to its wide range of sources and high reactivity [1]. Its oxidation product, 2,5-furandicarboxylic acid (FDCA), is a key monomer for the synthesis of poly (ethylene furandicarboxylate) (PEF). With its excellent gas barrier properties, mechanical properties, and degradability, PEF is recognized as an ideal substitute for petroleum-based polyethylene terephthalate (PET), so FDCA is listed by the US Department of Energy as one of the 12 most valuable bio-based platform compounds [2].

However, the current synthesis technology of FDCA still mainly relies on noble metal catalysis, electrocatalysis, and photocatalysis [3,4,5]. These methods generally have defects such as high cost, limited scalability, and harsh reaction conditions, which limit their industrial application prospects [6]. In contrast, biocatalysis technology shows significant advantages, especially in the oxidation system based on fungal laccase: it uses oxygen (O_2_) as an environmentally friendly terminal electron acceptor, produces only water as a reaction by-product, and the conditions are mild [7]. By introducing small molecular mediators (such as 2,2,6,6-tetramethylpiperidine-1-oxyl radical, TEMPO) to construct a laccase–mediator system (LMS), the catalytic efficiency of laccase on non-phenolic substrates (such as HMF) can be effectively improved, which provides an ideal path for the green oxidation of HMF [8].

Nevertheless, the industrial application of free laccase system still faces three key bottlenecks: the inherent structural vulnerability of enzyme molecules leads to poor operational stability; it has low tolerance of industrial-grade mechanical shear force; and the narrow pH range (4–6) and temperature window (20–40 °C) significantly increase the cost of process control. The continuous loss of enzymes caused by non-recyclable properties further increases production costs [9,10]. Although traditional immobilization techniques (such as physical adsorption or covalent cross-linking) aim to improve enzyme stability, they often lead to decreased enzyme activity or even inactivation due to improper carrier selection, limited enzyme conformation, or insufficient mass transfer efficiency, and still fail to fundamentally solve the ‘easy activation and difficult utilization’ problem of enzymes [11].

In 2012, the ‘enzyme–inorganic hybrid nanoflower’ strategy proposed by Ge et al. opened up a new way for enzyme immobilization. This method uses laccase itself as the nucleation site of copper phosphate crystal to induce self-assembly with Cu^2+^/PO_4_^3−^ system to form a three-dimensional porous flower-like structure [12]. In this nanoflower, the enzyme molecules are embedded in the inorganic crystal skeleton rather than simply coated or adsorbed, thus taking into account the high activity retention and structural stability. The unique open layered morphology of nanoflowers provides highly accessible enzyme active sites and excellent substrate diffusion channels, allowing the substrate molecules to quickly approach the reaction center, improving the conversion efficiency and inhibiting the occurrence of side reactions [13]. However, due to the lack of efficient separation methods, it is difficult to achieve convenient recovery and recycling in continuous flow operation and industrial scale-up process, which seriously restricts its practical application value.

In order to break through the above bottlenecks, this paper innovatively introduces ‘magnetic copper ions’ as the nucleation source, and proposes a composite immobilization strategy combining magnetic response regulation and spatial self-assembly. Different from the traditional copper source-induced crystallization methods, such as CuSO_4_ or CuCl_2_ [11], we used the metal chelating agent N-(phosphonomethyl) iminodiacetic acid (PMIDA) modified Fe_3_O_4_@SiO_2_ nanoparticles for the first time to directionally chelate Cu^2+^ to participate in the growth process of copper phosphate crystals in the form of magnetic response. This strategy not only endows the material with fast magnetic separation ability, but also significantly improves the controllability of the crystal structure and the spatial stability of the enzyme. The constructed magnetic core-induced copper phosphate-laccase nanoflower catalyst (MLac-NFs) significantly enhanced the thermal stability and pH adaptability of the enzyme through the core-shell synergistic structure, and inhibited the fluctuation of enzyme activity due to the change of reaction conditions, effectively prolonging the catalytic life. The platform shows high activity, super stability, and high recyclability in the catalytic reaction of HMF to FDCA, and constructs a new green catalytic path that takes into account both reaction efficiency and industrial applicability.

In this study, the comprehensive performance of MLac-NFs in the catalytic oxidation of HMF to FDCA was systematically evaluated, including catalytic activity, reaction kinetics, pH/temperature tolerance, cycle stability, and structural durability. The results show that the system exhibits significant application potential, which not only provides a solution with great industrial prospects for the green and efficient synthesis of FDCA but also establishes a universal optimization strategy for the design of high-performance enzyme catalytic systems suitable for continuous flow reactions and industrial amplification. This design fundamentally solves the core contradiction of “activity-stability-recyclability” in traditional enzyme immobilization through the synergy of biomimetic mineralization and magnetic engineering.

## 2. Materials and Methods

### 2.1. Chemicals and Reagents

Laccase was derived from laboratory-cultured *Trametes versicolor* strains and was used without prior purification, with a crude enzyme activity of 28.5 U mg^−1^. Potato dextrose broth and agar powder were purchased from Qingdao Science and Technology Industrial Park Haibo Biotechnology Co., Ltd. (Qingdao, China). Copper sulfate pentahydrate (CuSO_4_·5H_2_O), sodium citrate (C_6_H_5_Na_3_O_7_), disodium hydrogen phosphate dodecahydrate (Na_2_HPO_4_·12H_2_O), sodium dihydrogen phosphate dihydrate (NaH_2_PO_4_·2H_2_O), magnesium sulfate heptahydrate (MgSO_4_·7H_2_O), ammonium dihydrogen phosphate (NH_4_H_2_PO_4_), ferrous sulfate heptahydrate (FeSO_4_·7H_2_O), ammonium chloride (NH_4_Cl), glucose, concentrated phosphoric acid (H_3_PO_4_), sodium chloride (NaCl), potassium dihydrogen phosphate (KH_2_PO_4_), calcium chloride dihydrate (CaCl_2_·2H_2_O), ferric chloride hexahydrate (FeCl_3_·6H_2_O), ferrous chloride dihydrate (FeCl_2_·4H_2_O), ammonia (NH_3_·H_2_O), N-(phosphonomethyl) iminodiacetic acid (C_4_H_7_NO_4_), 3-chloropropyltrimethoxysilane (C_6_H_15_ClO_3_Si), tetraethyl orthosilicate (C_8_H_12_O_8_Si), methanol (CH_4_O), and ethanol (95%) were purchased from Guoyao Chemical Reagent Co., Ltd. (Shanghai, China). 2,2,6,6-tetramethylpiperidine-1-oxyl radical (TEMPO), 2,2′-hydrazine-bis (3-ethylbenzothiazoline-6-sulfonic acid) diamine (ABTS), and Coomassie brilliant blue G250 (BBG) were purchased from McLean Chemical Reagent Co., Ltd. (Shanghai, China). 5-Hydroxymethylfurfural (HMF), 2,5-diformylfuran (DFF), 5-formyl-2-furancarboxylic acid (FFCA), and 2,5-furandicarboxylic acid (FDCA) were purchased from Aladdin Chemical Reagent Co., Ltd. (Shanghai, China). All chemicals are analytical grade and can be used without further purification.

### 2.2. Preparation of MLac-NFs

Fe_3_O_4_@SiO_2_-PMIDA-Cu^2+^ was prepared according to the previous report [14]. FeCl_3_·6H_2_O and FeCl_2_·4H_2_O were mixed at a molar ratio of 2:1, with the total iron ion concentration in the system adjusted to 0.015 M. Under the protection of a nitrogen atmosphere and constant stirring, the solution was heated to 80 °C. Then, 10 mL of 25% (*w*/*v*) ammonia aqueous solution was added, along with 0.0003 M sodium citrate as a surface modifier, and the reaction was sustained for 30 min. The resulting Fe_3_O_4_ particles were collected and washed with Millipore water (Burlington, MA, USA). The Fe_3_O_4_ nanoparticles were uniformly dispersed in 50 mL of an ethanol/water mixed solvent (*v*/*v*, 3:1), followed by the addition of 2.8 mL of tetraethyl orthosilicate (TEOS), and stirring was continued for 30 min. Subsequently, 2.5 mL of ammonia water was added as a catalyst, and continuous stirring was maintained for 12 h under constant temperature conditions. The obtained magnetic silica composite nanomaterials (Fe_3_O_4_@SiO_2_) were collected and washed multiple times with absolute ethanol and Millipore water in sequence. A total of 30 mg of Fe_3_O_4_@SiO_2_ composite nanomaterials were uniformly dispersed in 5 mL of Millipore water, subjected to ultrasonic treatment for 60 min, and then transferred to a three-necked flask. Under mechanical stirring at 200 rpm, 5 mL of a 15 mg mL^−1^ aqueous solution of N-(phosphonomethyl)iminodiacetic acid (PMIDA) was added, and the pH of the reaction system was adjusted to 10 using 2.5 M ammonia aqueous solution. The entire reaction was carried out at room temperature for 12 h, and the product was repeatedly washed with distilled water until the pH of the filtrate reached neutrality, finally obtaining PMIDA-modified magnetic silica composite nanomaterials (Fe_3_O_4_@SiO_2_-PMIDA). An appropriate amount of Fe_3_O_4_@SiO_2_-PMIDA nanomaterials was uniformly dispersed in 5 mL of ultrapure water. After continuous mechanical stirring for 30 min, a 500 mM aqueous solution of copper sulfate pentahydrate (CuSO_4_·5H_2_O) was slowly added dropwise. The reaction system was stirred continuously for 3 h under constant stirring conditions to prepare copper ion-chelated magnetic silica composite nanomaterials (Fe_3_O_4_@SiO_2_-PMIDA-Cu^2+^). Preparation of MLac-NFs: 10 mL of laccase solution (25 μg mL^−1^) was accurately transferred into a 100 mL conical flask, and 40 mL of PBS buffer (pH 7.4, 10 mM) was added and mixed well. A certain amount of uniformly dispersed Fe_3_O_4_@SiO_2_-PMIDA-Cu^2+^ suspension (5 mg mL^−1^, containing 500 mM CuSO_4_·5H_2_O) was added and mixed in a 120 rpm shaker for 5 min. After incubation at 4 °C for 24 h, MLac-NFs were obtained and collected by magnetic separation. After washing with distilled water 3 times, it was dispersed in PBS buffer and stored at 4 °C.

### 2.3. Characterization of Nanoflowers

The morphology of MLac-NFs was observed by an S-4800 cold field emission scanning electron microscope (SEM) produced by the Hitachi company in Tokyo, Japan. The functional groups of the carrier were analyzed by an FTIR-8400 S Fourier transform infrared spectrometer (Shimadzu, Kyoto, Japan). The magnetic properties of the samples were tested by a Lake Shore 7404 vibrating sample magnetometer (Westerville, OH, USA) at room temperature. The crystal structure was determined by a Malvern Panalytical multifunctional powder X-ray diffractometer (Worcestershire, UK). The diffraction pattern was obtained at a speed of 0.1° per second in a continuous scanning mode in the range of 5–70°.

### 2.4. Determination of Laccase Activity

The activity of MLac-NFs was determined using ABTS as a substrate [15]. A total of 100 μL of MLac-NFs suspension with a concentration of 25 μg mL^−1^ (this concentration refers to the protein concentration after laccase is immobilized on the magnetic material) and free laccase with the equivalent protein concentration were, respectively, added to the mixture of 0.5 mM ABTS and sodium tartrate buffer (pH 4.0, 0.1 M), and the total volume was adjusted to 1 mL. The mixture was shaken at 26 °C for 3 min, and the suspension was separated. The absorbance of the supernatant was measured at 420 nm using a UV-2000 spectrophotometer (UV-Vis Spectrophotometer, Shimadzu, Kyoto, Japan). The definition of enzyme activity: the amount of laccase required to oxidize 1 μM ABTS in 1 min is an enzyme activity unit (U). The calculation formulas for enzyme activity and relative enzyme activity are as follows:

$$U/L=\frac{{∆\mathrm{OD}}_{420}\times 10^{6}\times V_{s}\times n}{\varepsilon \times ∆T\times V_{t}\times d}$$

$$Relative\;activity(\%)=\frac{Enzyme\;activity\;under\;specified\;conditions\;(pH/temperature)}{Enzyme\;activity\;under\;optimal\;conditions\;(pH/temperature)}$$

where ΔOD_420_ is the difference in absorbance between the sample and the blank control at 420 nm; ε is the molar extinction coefficient of ABTS (36,000 L·mol^−1^·cm^−1^); Vt and Vs (L) represent the total volume of the reaction system and the volume of laccase, respectively; ΔT (min) is the reaction time; d is the optical path length (1 cm); and n is the dilution factor of the crude laccase.

### 2.5. Determination of Apparent Kinetic Constants of Laccase and MLac-NFs

ABTS was dissolved in sodium tartrate buffer with a pH value of 4.0, and reaction solutions with concentrations of 0.015625 mM, 0.03125 mM, 0.0625 mM, 0.125 mM, 0.25 mM, and 0.5 mM were prepared, respectively. Subsequently, the apparent kinetic constants (*K_m_*) and the maximum reaction rate (*V_m_*) of the enzymatic reaction of free laccase and Mlac-NFs were determined. The experiment was conducted based on the same amount of laccase protein, and a nonlinear regression equation fitting analysis was performed using the Michaelis–Menten plot method [16]. The calculation formula is as follows:

$$V=\frac{V_{max}[S]}{K_{m}+[S]}$$

where *V* is the rate of enzymatic reaction (mM); *K_m_* is the Michaelis constant (mM); *V_m_* is the maximum reaction rate (mM min^−1^); and *S* is the substrate concentration (mM). The apparent kinetic parameters *K_m_* and *V_m_* were calculated according to the slope and intercept, respectively.

### 2.6. Effects of pH and Temperature on Laccase Activity

The effects of different pH (3.0, 4.0, 5.0, 6.0, 7.0, and 8.0) on laccase activity were investigated at 30 °C, and the effects of different temperatures (20, 30, 40, 50, 60, and 70 °C) on laccase activity were investigated at the optimum pH. The experiment was conducted based on the same amount of laccase protein.

### 2.7. Storage Stability Experiment

MLac-NFs were stored in PBS (10 mM, pH 7.4) at 4 °C and 25 °C for 30 days. The residual activity of the sample was measured at the same time interval, and the initial activity was determined to be 100% to evaluate its storage stability. The experiment was conducted based on the same amount of laccase protein.

### 2.8. HMF Conversion

The mechanism of its catalytic oxidation of HMF to FDCA is shown in Figure S1. The transformation process proceeds as follows: HMF and TEMPO are introduced into 1 mL of PBS buffer containing magnetic hybrid nanoflowers (MLac-NFs) at a concentration of 8 mg mL^−1^, resulting in their final concentrations being adjusted to 10 mM and 30 mM, respectively. Under continuous stirring, aeration was conducted for 20 min per hour. The samples after the reaction were diluted with PBS buffer to 50 times the original concentration, filtered through a 0.22 μm filter membrane, and analyzed by high-performance liquid chromatography (Agilent Infinity II LC, Santa Clara, CA, USA) equipped with a UV detector and C18 column (Diamonsil). The analysis conditions were as follows: mobile phase: 0.1% trifluoroacetic acid aqueous solution and acetonitrile, volume ratio of 85:15, column temperature of 40 °C, flow rate of 0.15 mL min^−1^, and injection volume of 20 μL. The detection wavelength spectrum of HMF, DFF, FFCA, and FDCA (Figure S2), the calibration curves of HMF, DFF, FFCA, and FDCA (Figure S3), the retention times of each compound (Figure S4), and the detection wavelengths (Table S1). A single-factor experimental design was employed to investigate the effects of pH value, temperature, and TEMPO concentration on the conversion of HMF to FDCA. During the whole experiment, the reaction conditions were pH (4.0, 6.0, and 8.0), reaction temperature (20, 30, 40, 50, 60, and 70 °C), and TEMPO concentration (10, 15, 20, 25, 30, 35, and 40 mM). The calculations for the yield and relative yield of FDCA are as follows:

$$Yield=\frac{Mol\;amount\;of\;actually\;produced\;FDCA}{Mol\;amount\;of\;initial\;reaction\;substrate\;HMF}$$

$$Relative\;yield=\frac{Mol\;amount\;of\;actually\;produced\;FDCA\;in\;the\;nth\;cycle}{Mol\;amount\;of\;actually\;produced\;FDCA\;in\;the\;first\;cycle}$$

### 2.9. Total Turnover Number

The Total Turnover Number (TTN) is a core indicator for evaluating the longevity and economic efficiency of catalysts, particularly biocatalysts. It is defined as the total number of substrate molecules that a single catalyst molecule (or active site) can convert before becoming inactivated. The calculation formula is as follows:

$$TNT=\frac{Total\;amount\;of\;converted\;substrate\;(mol)}{Total\;amount\;of\;active\;sites\;in\;the\;catalyst\;(mol)}$$

## 3. Results and Discussion

### 3.1. Structural Characterization of MLac-NFs

#### 3.1.1. SEM

**Figure 1:**
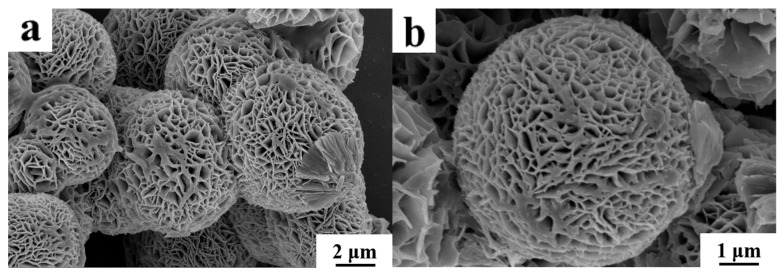
SEM images of MLac-NFs at different Sizes. (**a**) 2 μm, and (**b**) 1 μm.

The morphology of MLac-NFs was characterized by SEM. As shown in Figure 1, MLac-NFs were porous spherical particles with a particle size of about 8–14 μm. The porous structure was formed by self-assembly of flaky petal units with a thickness of about 18–23 nm. It was noted that magnetic nanoparticles were attached to the surface of these petals, and the particles had obvious aggregation. The particle aggregation led to a decrease in the internal voids of the nanoflowers, and the overall structure was more compact [17].

#### 3.1.2. FT-IR

**Figure 2:**
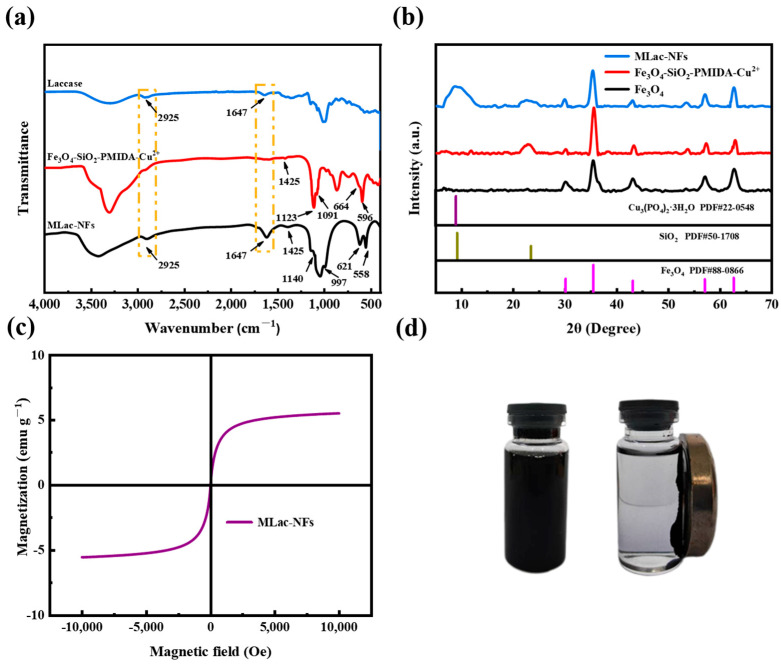
MLac-NFs characterization (**a**) FT-IR, (**b**) XRD, (**c**) VSM, and (**d**) magnetic adsorption.

In order to confirm whether laccase was successfully immobilized on MLac-NFs, Laccase, Fe_3_O_4_@SiO_2_-PMIDA-Cu^2+^, and MLac-NFs were analyzed by infrared spectroscopy. As shown in Figure 2a, in the infrared spectrum of Fe_3_O_4_@SiO_2_-PMIDA-Cu^2+^, the strong absorption peaks at 596 cm^−1^, 1091 cm^−1^, and 1123 cm^−1^ corresponded to Fe–O vibration, Si–O–Si stretching vibration peak, and Si–O–P bond stretching vibration on the PMIDA molecule on the surface of magnetic nanoparticles, indicating that SiO_2_ had been gradually successfully modified on Fe_3_O_4_. The weak absorption peak at 1425 cm^−1^ corresponded to the C–O–H in-plane bending vibration of PMIDA [18]. The absorption peak at 1647 cm^−1^ was due to the influence of-COOH groups in PMIDA molecules on the surface of magnetic nanoparticles and –OH hydrogen bonds in H_2_O, resulting in its shift. Therefore, FT-IR spectra confirmed the successful connection between PMIDA and the surface of SiO_2_-coated magnetic nanoparticles [19]. The absorption peak at 1647 cm^−1^ represented the –CONH amide band in laccase, while the peak at 2925 cm^−1^ was attributed to the C–H symmetric stretching vibration of CH_2_ in laccase molecules [20], both of which existed in MLac-NFs. In addition, the characteristic absorption peaks at 621 cm^−1^ and 558 cm^−1^ in MLac-NFs were O–P–O bending vibration; the peak at 997 cm^−1^ corresponded to the asymmetric stretching vibration of PO_4_^3−^ [21]. These results further confirmed the presence of laccase on MLac-NFs.

#### 3.1.3. XRD

In order to clarify the inorganic components in MLac-NFs, Fe_3_O_4_, Fe_3_O_4_@SiO_2_-PMIDA-Cu^2+^, and MLac-NFs were analyzed by X-ray diffraction (XRD) (Figure 2b). By comparing with standard powder diffraction cards (JCPDS, PDF#88-0866 (Fe_3_O_4_), PDF#50-1708(SiO_2_), PDF#22-0548(Cu_3_(PO_4_)_2_·3H_2_O)), the XRD patterns of Fe_3_O_4_, SiO_2_, and Cu_3_(PO_4_)_2_·3H_2_O were well matched with those of Fe_3_O4, Fe_3_O_4_@SiO_2_-PMIDA-Cu^2+^ and MLac-NFs. The highest peaks of SiO_2_ (9.14°) and Cu_3_(PO_4_)_2_·3H_2_O (8.92°) were superimposed [22]. This is due to the existence of a phase with a similar crystal plane spacing between SiO_2_ and Cu_3_(PO_4_)_2_·3H_2_O. XRD analysis proved that SiO_2_ was successfully coated on the surface of Fe_3_O_4_.

#### 3.1.4. VSM

In order to verify the recovery performance of the material, a vibrating sample magnetometer (VSM) was used to determine the hysteresis loop diagram of MLac-NFs. As shown in Figure 2c, MLac-NFs had superparamagnetic remanent magnetization close to zero, with a maximum saturation strength of 5.52 emu g^−1^. The decrease in saturation magnetization with the synthesis of materials may be due to the gradual increase in Fe_3_O_4_ loading materials and the formation of magnetization hindrance with laccase, which resulted in the decrease in saturation magnetization of the final product MLac-NFs [23]. Figure 2d shows the recovery effect of MLac-NFs under an external magnetic field. Although the saturation magnetization decreased, ordinary magnets can still be used for recovery to facilitate recycling.

### 3.2. Enzymatic Properties of MLac-NFs

#### 3.2.1. Effect of pH on MLac-NFs Enzyme Activity

**Figure 3:**
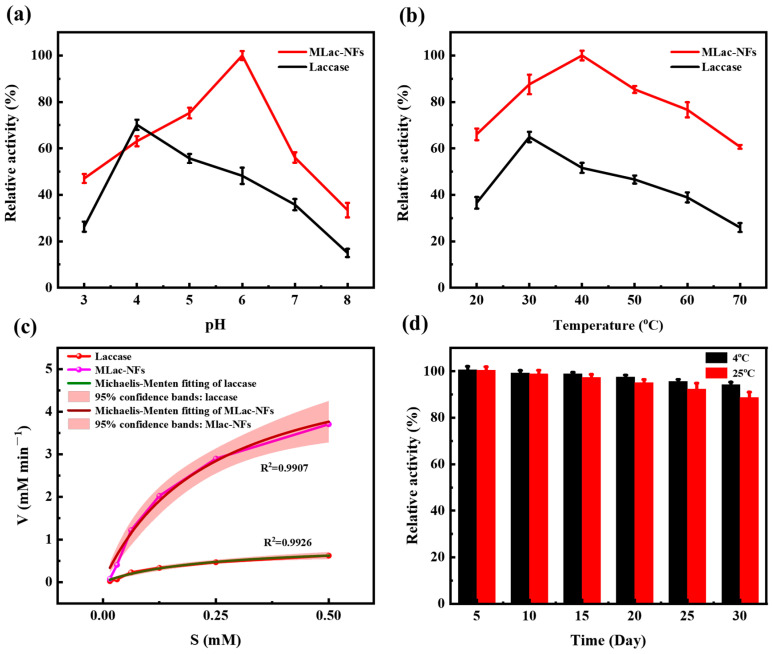
Enzymatic properties of MLac-NFs: (**a**) effect of pH on enzyme activity, (**b**) effect of temperature on enzyme activity, (**c**) apparent kinetics, and (**d**) storage stability. Data represent mean values ± SD of triplicates.

The pH-dependent analysis (Figure 3a) showed that the optimum pH of MLac-NFs was 6.0, at which the enzymatic activity of MLac-NFs reaches 9.25 U mL^−1^. The effect of pH on enzymatic activity was analyzed with the maximum activity set as 100%. The relative enzyme activity increased significantly with the increase in pH in the range of pH 3.0 to 6.0. In the range of pH 6.0 to 8.0, it gradually decreased with the increase in pH. At the optimal pH of 6.0, the relative enzyme activity of MLac-NFs reached its peak. The peak activity was 2.18 times and 5.26 times higher than that at pH 3.0 and pH 8.0, respectively. Compared with the free laccase from Trametes versicolor, the immobilized MLac-NFs showed a wider pH range, and the optimal activity region shifted to the alkaline direction. Nevertheless, its highest activity point (optimum pH 6.0) was still in the acidic range [24].

#### 3.2.2. Effect of Temperature on MLac-NFs Enzyme Activity

As shown in Figure 3b, the optimum catalytic temperature of MLac-NFs was 40 °C higher than 30 °C of free laccase. In the range of 20–45 °C, the activity of MLac-NFs increased slowly with increasing temperature. In the high temperature range of 50–70 °C, it still maintained high activity, indicating that its thermal stability was significantly enhanced. This performance improvement can be attributed to the multiple stabilizing effects of the immobilized structure on laccase molecules. First, the core-shell structure constructed by the Fe_3_O_4_ magnetic core and the Cu_3_(PO_4_)_2_·3H_2_O flower-like crystal shell synergistically inhibits the conformational fluctuation of the enzyme through the spatial confinement effect and reduces the high-temperature-induced conformational relaxation and inactivation. Second, the multi-site chelation between Cu^2+^ in the crystal shell and the functional groups on the surface of the enzyme enhances the structural rigidity of the molecule and improves the conformational retention ability under thermal disturbance [25].

In addition, the conformation of the enzyme molecule tends to be compact after immobilization, and the configuration stability of the substrate binding site was enhanced. This may have led to a slight increase in the activation energy required to overcome when the substrate is bound to the active center, thereby delaying the deactivation rate during thermal activation. This weak increase in activation energy inhibited the non-selective side reactions at high temperatures to a certain extent, and helped to maintain catalytic specificity and long-term operational stability [26]. After immobilization, the temperature window of free laccase is broadened, thereby enhancing its application prospects.

#### 3.2.3. Apparent Kinetic Constants of MLac-NFs

In order to further demonstrate the catalytic advantages of the immobilized system, the researchers performed apparent kinetic analysis using the Michaelis–Menten equation. As shown in Figure 3c, the enzymatic reaction rate saturates with increasing substrate concentration. The coefficient of determination (R^2^) values for both free laccase and MLac-NFs were close to 1, indicating high accuracy of the experimental data. To confirm that the fitting curve remained valid at high substrate concentrations, the researchers measured the reaction rates at 0.75 mM and 1.5 mM substrate concentrations and performed nonlinear fitting. As shown in Figure S6, the reaction rates approached saturation under high substrate concentrations. As shown in Table 1, under the condition of the same enzyme amount, the catalytic behavior was fundamentally improved after immobilization: The apparent *K_m_* value of MLac-NFs was reduced by 38.3% (*p* < 0.001), which reflected the substrate affinity enhanced by the optimized ABTS diffusion in the hierarchical pores of nanoflowers. At the same time, the apparent *V_max_* value of MLac-NFs increased by 439.6% (*p* < 0.001). It showed that the electron transfer efficiency mediated by Cu^2+^ coordination between adjacent laccase molecules is improved. This synergistic kinetic enhancement not only alleviates mass transfer bottlenecks but also accelerates the catalytic process, which was highly consistent with the observed activity improvement [27], confirming the structural advantages of the magnetic hybrid nanoflower structure in optimizing enzyme catalytic performance.

#### 3.2.4. Storage Stability of MLac-NFs

The storage stability of MLac-NFs was directly related to their later recycling. In this study, the storage stability of MLac-NFs was investigated at 4 °C and 25 °C, respectively. As shown in Figure 3d, MLac-NFs can still maintain 94.26% of their initial enzyme activity after storage at 4 °C for 30 days, and can maintain 88.75% of their initial enzyme activity after storage at 25 °C for 30 days. Free laccase had a high content of histidine residues in the laccase molecule, and the active center contained histidine residues. These residues were partially exposed to the outside of the molecule, and the enzyme activity decreased significantly over time. After the material loading treatment, the Cu^2+^ chelated by the carrier can affinity adsorb these exposed histidine residues and interact with multiple sites, thereby maintaining the spatial structure of the laccase active center stable [28].

### 3.3. MLac-NFs Catalyze the Oxidation of HMF to FDCA

In the laccase–TEMPO synergistic catalytic system, laccase first activates O_2_ and reduces it to water, while oxidizing the mediator TEMPO to the highly active TEMPO^+^. As the actual oxidizing agent, TEMPO^+^ attacks HMF molecules in a stepwise manner: it first selectively oxidizes the hydroxymethyl group to form the aldehyde intermediate DFF. Subsequently, in an aqueous environment, the aldehyde group of DFF hydrates to form a geminal diol, which is then oxidized by TEMPO^+^ to a carboxyl group, generating FFCA. Finally, the aldehyde group of FFCA undergoes the same hydration–oxidation process to be converted into a carboxyl group, forming the target product FDCA. The reduced TEMPO generated in the reaction is re-oxidized by laccase to regenerate TEMPO^+^, thus forming an efficient catalytic cycle with oxygen as the terminal electron acceptor. Schematic diagram of efficient conversion of HMF to FDCA by MLac-NFs with TEMPO as the mediator (Figure S1).

#### 3.3.1. Effect of pH on the Conversion of HMF to FDCA over MLac-NFs Catalyst

**Figure 4:**
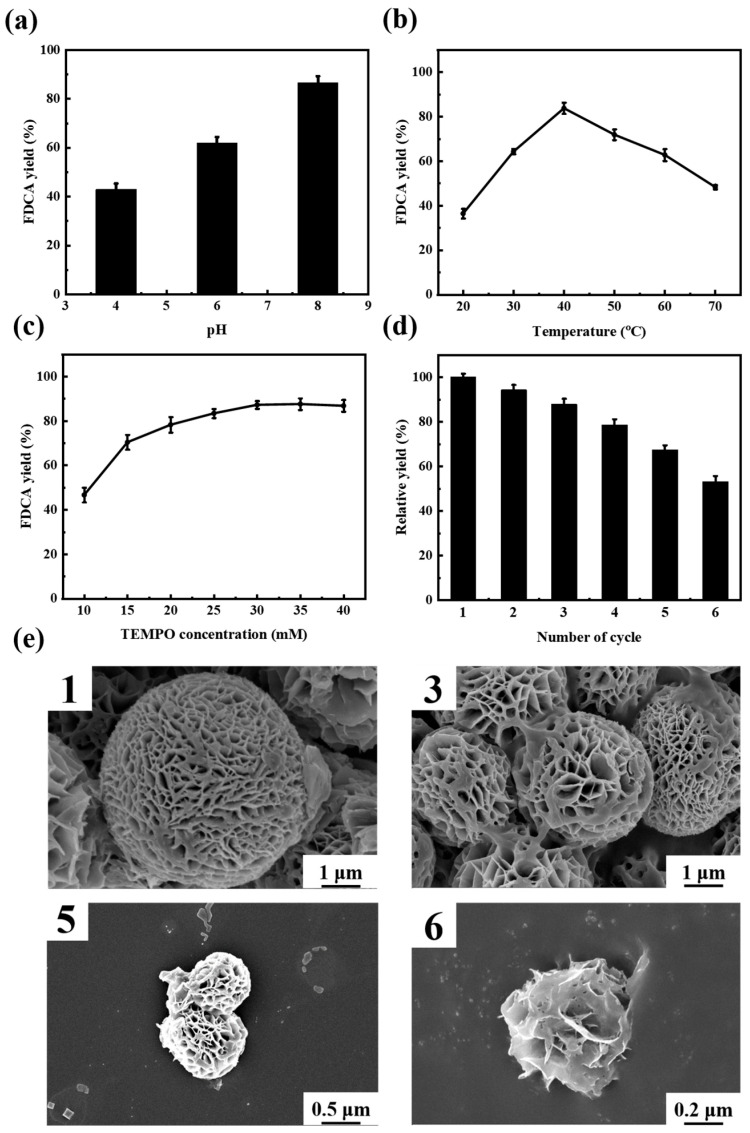
The catalytic performance of MLac-NFs for HMF (**a**) pH, (**b**) temperature, (**c**) TEMPO concentration, (**d**) recyclability, and (**e**) SEM image after cyclic reaction (Numeric value indicates cycle number). Data represent mean values ± SD of triplicates.

The fluctuation of catalytic activity caused by environmental changes was relatively stable after laccase was loaded on the carrier. The results are shown in Figure 4a; the yield of FDCA is only 42.37% at pH 4.0, reaches 63.26% at pH 6.0, and rises to as high as 86.73% at pH 8.0. The reason for this is that as the reaction proceeds, the functional group changes from a hydroxyl group to an aldehyde group and finally to a carboxyl group; the pH gradually decreases, and then reaches its optimal pH. Moreover, the hybrid nanoflower structure formed by MLac-NFs was more compact, and most magnetic nanoparticles were encapsulated inside the nanoflowers, which reduced the coverage of magnetic nanoparticles on the surface-active sites of nanoflowers [29].

This change was mainly due to the regulation of the immobilization process on the enzyme conformation and local environment. On the one hand, the laccase in the nanoflower structure was embedded in the inorganic crystal skeleton, and its spatial rigidity was enhanced, which reduced the sensitivity to the fluctuation of proton concentration [30]. On the other hand, the introduction of magnetic nanoparticles regulated the microenvironment around the enzyme through surface functional groups, forming a relatively buffered reaction interface, which further stabilized the active state of the enzyme. Combined with the good mass transfer channel provided by the flower-like structure, the diffusion efficiency and enzyme binding ability of the substrate at higher pH were significantly improved, and the overall synergy promoted the optimal pH to move up and slowed down the fluctuation of the activity with pH [31]. This result not only improved the stability of MLac-NFs in complex reaction environments but also provided a basis for their application in practical industrial processes.

#### 3.3.2. Effect of Temperature on the Oxidation of HMF to FDCA over MLac-NFs Catalyst

Temperature significantly affected the activity, stability, and reaction rate of the loaded enzyme. According to the Arrhenius equation, the reaction rate usually increases with an increase in temperature. In application, the reaction temperature needed to be controlled within the optimal range to maintain the efficiency and stability of the enzyme [32]. As shown in Figure 4b, the optimum catalytic temperature of MLac-NFs was 40 °C, and the conversion of HMF was 100% and the yield of FDCA was 83.74% at 40 °C. The yield at the optimal temperature is 2.16 times that at 20 °C and 1.72 times that at the highest temperature. The relatively high yield of FDCA under high-temperature conditions indicates that the laccase-magnetic nanofiber (MLac-NFs) catalyst prepared by loading onto magnetic materials has the potential for application in high-temperature environments [33].

#### 3.3.3. Effect of TEMPO Concentration on the Catalytic Oxidation of HMF to FDCA by MLac-NFs

The conversion of HMF to FDCA was completed by the electron transfer generated by the reaction between laccase and the mediator (TEMPO) [34]. The yield of FDCA under different TEMPO concentrations is shown in Figure 4c. The yield of FDCA increases with the rise in TEMPO concentration, reaching the optimal level of 86.82% when the concentration is 30 mM. This trend indicated that when the concentration of TEMPO was low, the lack of an electron acceptor limited the reaction rate; when the concentration increased to match the fixed enzyme amount, the system reached electron transfer rate saturation, and further addition of TEMPO no longer improved the yield of FDCA. Therefore, the optimal concentration reflected the synergistic balance between the available active sites on the surface of MLac-NFs and the mediator.

#### 3.3.4. Reusability of MLac-NFs

The reusability of the immobilized enzyme was one of the important advantages of the application of the immobilized enzyme in the biocatalysis industry. The magnetic laccase catalyst was quantitatively separated from the reaction mixture by an external magnetic field, and then washed and reused in subsequent cycles under the same reaction conditions. As shown in Figure 4d, the yield of FDCA can still reach 53.26% after six cycles, indicating that it can be successfully recycled. The reusability of MLac-NFs may be attributed to the multi-point bonding between laccase molecules and magnetic carriers, which improved the stability of the laccase active conformation [35]. As shown in Figure 4e, SEM analysis of the material after recycling showed that MLac-NFs began to dissociate gradually with the increase in the number of cycles. The leakage of immobilized laccase induced by the dissociation of MLac-NFs was verified by the color development when the reaction solution was mixed with ABTS, a substrate for laccase activity determination. In the formation mechanism of MLac-NFs, the laccase protein functions as a bridging molecule. The leakage of laccase results in the dissociation of MLac-NFs, which is consistent with the SEM observations. As laccase is dissolved into the reaction solution, the loss of laccase further leads to a reduction in the conversion efficiency of HMF to FDCA. In summary, free laccase can improve the ability of laccase to cope with environmental changes after being loaded on magnetic carriers, reduce its denaturation, and may accelerate the reaction [36].

#### 3.3.5. MLac-NFs Successfully Achieved the Complete Conversion of HMF to FDCA

**Figure 5:**
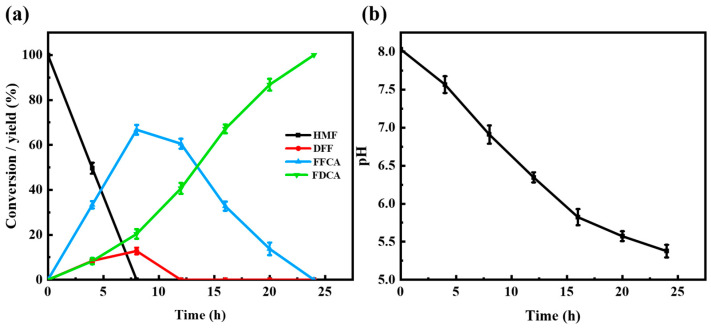
(**a**) Time profile of HMF catalytic oxidation by MLac-NFs, and (**b**) graph of pH variation with reaction time. Data represent mean values ± SD of triplicates.

Under optimal reaction conditions, the kinetic process of HMF to FDCA catalyzed by MLac-NFs and the pH change of the reaction solution are shown in Figure 5. As the reaction progressed, HMF was continuously consumed, and the intermediate product and the final product were increasing. The added value of intermediate product DFF was very low, indicating that it only played a transitional role. When the reaction was carried out for 8 h, HMF was completely transformed, the content of intermediate product DFF began to decrease gradually, and the content of FFCA also began to decrease after reaching a maximum of 68.3%. After 12 h, only FFCA and FDCA were left in the reaction solution. The conversion trend gradually decreased during the conversion of FFCA to FDCA, because the pH of the functional group decreased from 8.03 to 5.45 as the reaction progressed, and the catalytic activity of MLac-NFs increased first and then decreased with the decrease in pH. When the reaction reached 24 h, the reaction product and intermediate product were completely converted to FDCA. To investigate whether magnetic nanomaterials possess the ability to catalytically oxidize HMF, a catalytic comparison was conducted between magnetic nanomaterials and MLac-NFs. As shown in Figure S7, only the reaction substrate HMF was present in the reaction over time, indicating that magnetic nanomaterials do not have the capability to catalyze HMF. The conversion of HMF to FDCA is attributed to the action of the laccase–TEMPO mediator system.

#### 3.3.6. Total Turnover Number (TTN) Analysis

The Total Turnover Number (TTN) is the most direct indicator of a catalyst’s stability and resistance to deactivation. A high TTN indicates that the catalyst can maintain activity over an extended period under reaction conditions, which is crucial for processes requiring long-term operation or handling feedstocks that may contain inhibitors. In industrial applications, a high TTN means that each catalyst molecule can convert more substrate, thereby significantly reducing the catalyst cost per unit product. The TTN of MLac-NFs under mild conditions reaches 1.32 × 10^4^, achieving a TTN level comparable to that of noble metal catalysts (10^4^–10^5^), with significant improvements in cost and safety.

#### 3.3.7. Catalytic Performance Comparison

To provide contextual support for the research in this paper, a comparative table analyzing catalytic performance encompassing substrate concentration, yield, and reaction time has been established against traditional systems, including chemical, electrochemical, photocatalytic, and biocatalytic approaches, as presented in Table 2. As indicated in the table, the catalytic process described in this study exhibits a relatively high substrate concentration, with a yield reaching 100% within a 24 h reaction period. Immobilized enzyme technology enables the transformation of enzymatic catalysis into a system characterized by easy separation, multiple reusability, enhanced operational stability, and more feasible continuous production. Simultaneously, it circumvents the limitations of alternative catalytic systems: the challenges in recovering homogeneous catalysts, the high costs associated with precious metals, and the harsh reaction conditions (high temperature/pressure, strong acids/alkalis, organic solvents) in chemical catalysis; potential issues of insufficient selectivity or equipment dependence in photo/electrocatalysis; and the complexity, excessive by-products, and difficult separation/purification of whole-cell systems. Overall, this approach offers a more environmentally benign, economically efficient, and highly specific catalytic solution.

## 4. Conclusions

Based on the strategy of ‘magnetic copper ion-induced mineralization’, this study constructed a laccase immobilization platform, MLac-NFs, with three-in-one performance (high activity, strong stability, and magnetic recovery). The directional chelation of Cu^2+^ was achieved by PMIDA-modified Fe_3_O_4_@SiO_2_, and the copper phosphate crystal was induced to self-assemble to form a flower-like structure with laccase as the nucleation site, which took into account the enzyme activity retention and structural stability. The porous layered morphology of the nanoflowers provides an excellent substrate diffusion path, and the magnetic core endows it with efficient recycling ability. The catalytic performance test showed that MLac-NFs could efficiently and selectively oxidize HMF to FDCA with the assistance of TEMPO. While improving thermal stability and pH adaptability, the catalytic life was significantly prolonged and reused many times. This strategy overcomes the problem of “stability-activity-recyclability“ seen in traditional immobilization systems.

## Figures and Tables

**Table 1:** Enzymatic apparent kinetics of free laccase and MLac-NFs.

Apparent *K_m_* Parameters	Laccase	MLac-NFs	* p
*K_m_* (mM)	1.75 ± 0.21	1.08 ± 0.09	<0.001
*V_max_* (mM min^−1^)	2.9 ± 0.34	12.75 ± 1.02	<0.001
*V_max_*/*K_m_* (min^−1^)	1.66 ± 0.18	11.8 ± 0.95	<0.001

Data are presented as the mean ± standard deviation (SD) of three independent experiments; paired *t*-test was performed with a significance level of α = 0.05. * denotes statistical significance.

**Table 2:** Catalytic conversion of HMF to FDCA by different methods.

Catalytic Method	Catalyst	HMF (mM)	Time (h)	FDCA Yield
Electrocatalysis [37]	50-NiN/GO-Ni-Foam (50 mg)	5	10	86.9 ± 4.1%
Photocatalysis [38]	30%FePc-Au/TiO_2_ (50 mg)	0.1	15	97%
Mon-atomic catalysis [39]	Co–N/F_1_ (50 mg)	0.2	3	99.20%
Biocatalysis [40]	TvGLOX (2 μM)	10	24	99%
Cascade Catalysis [41]	*T. reesei* cell (1 g L^−1^), Laccase(2.5 μM)	8	80	88%
Immobilized enzyme [42]	AAO (3 μM), HP-7@Fe_3_(PO_4_)_2_ (5 μM)	5	48	100%
This study	MLac-NFs (8 mg)	10	24	100%

## Data Availability

The original contributions presented in this study are included in the article/Supplementary Material. Further inquiries can be directed to the corresponding author.

## Supplementary Materials

The following supporting information can be downloaded at: https://www.mdpi.com/article/10.3390/ma18163780/s1, Figure S1: Schematic diagram of efficient conversion of HMF to FDCA by MLac-NFs with TEMPO as the mediator; Figure S2: Wavelength spectra for HMF, DFF, FFCA, and FDCA detection; Figure S3: Calibration curves (a) HMF, (b) DFF, (c) FFCA, and (d) FDCA; Figure S4: Retention times of each compound (a) HMF, (b) DFF, (c) FFCA, and (d) FDCA; Figure S5: Retention times of substrate, intermediates, and product; Figure S6: Enzymatic Kinetic; Figure S7: Magnetic material catalyzed oxidation of HMF; Table S1: Retention times and detection wavelengths of each compound.
