# Supplementary material for: Efficient Conversion of 5-Hydroxymethylfurfural to 2,5-Furandicarboxylic Acid by the Magnetic Laccase Nanoflowers-2,2,6,6-Tetramethylpiperidin-1-Oxyl System
Source: Materials (Basel). 2025 Aug 12;18(16):3780. doi: 10.3390/ma18163780 (PMC12542789; doi:10.3390/ma18163780)
Supplement: Supplementary file 1 [file materials-18-03780-s001.zip › materials-3775942-supplementary.pdf]

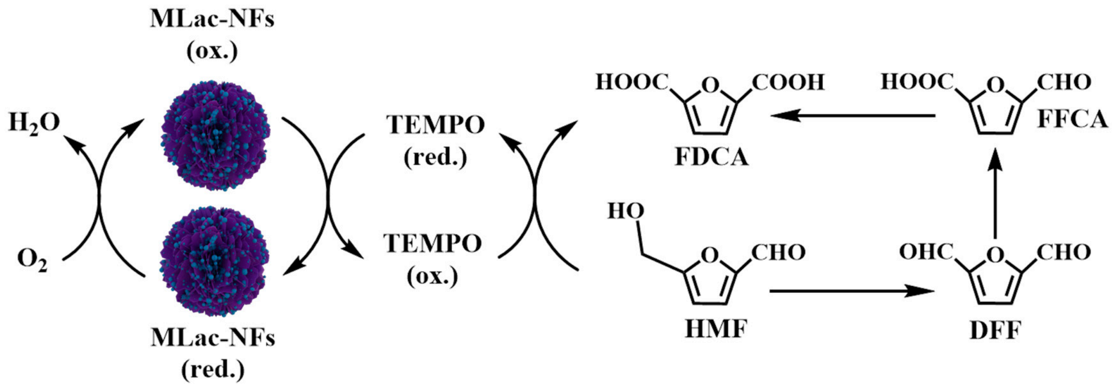

Figure S1. Schematic diagram of efficient conversion of HMF to FDCA by MLac-NFs with TEMPO as the mediator.

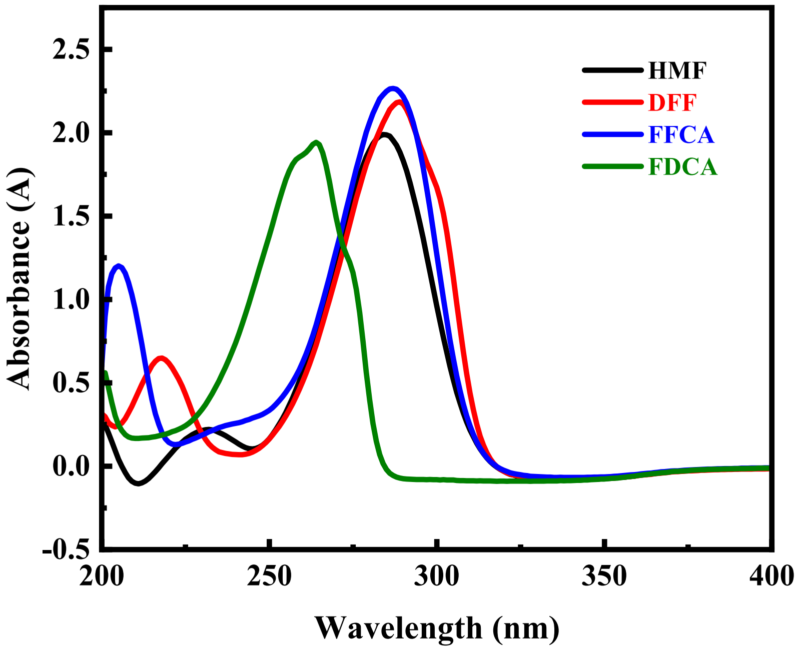

Figure S2. Wavelength spectra for HMF, DFF, FFCA, and FDCA detection.

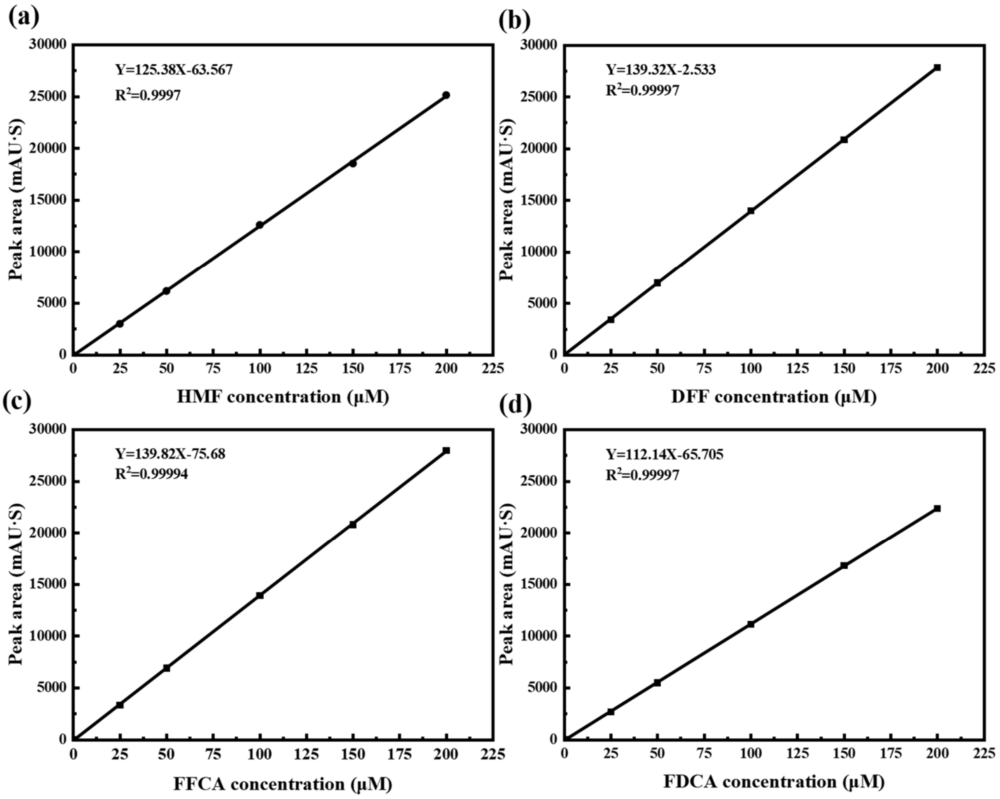

Figure S3. Calibration curves (a) HMF, (b) DFF, (c) FFCA, and (d) FDCA.

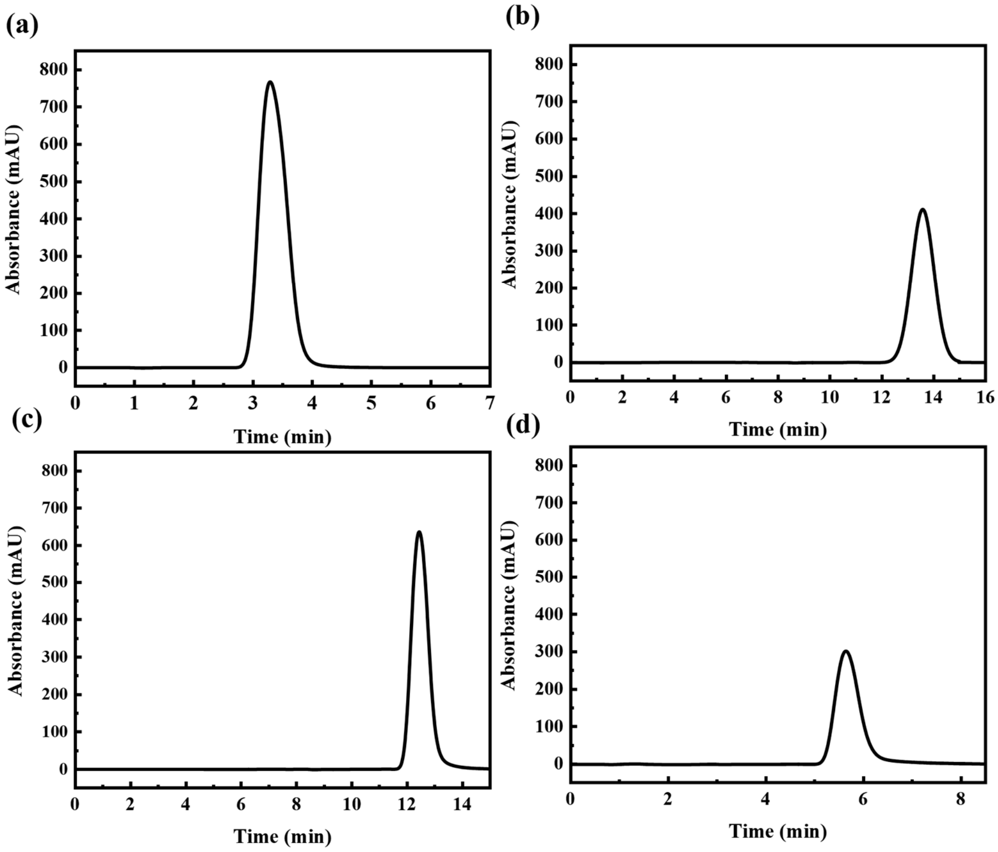

Figure S4. Retention times of each compound (a) HMF, (b) DFF, (c) FFCA, and (d) FDCA.

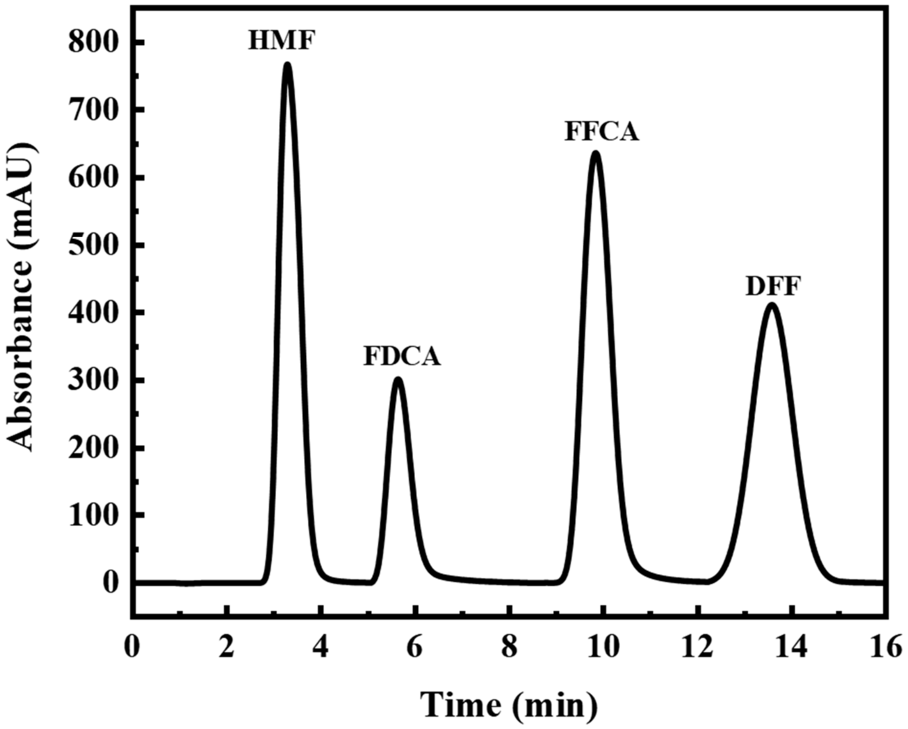

Figure S5. Retention times of substrate, intermediates, and product.

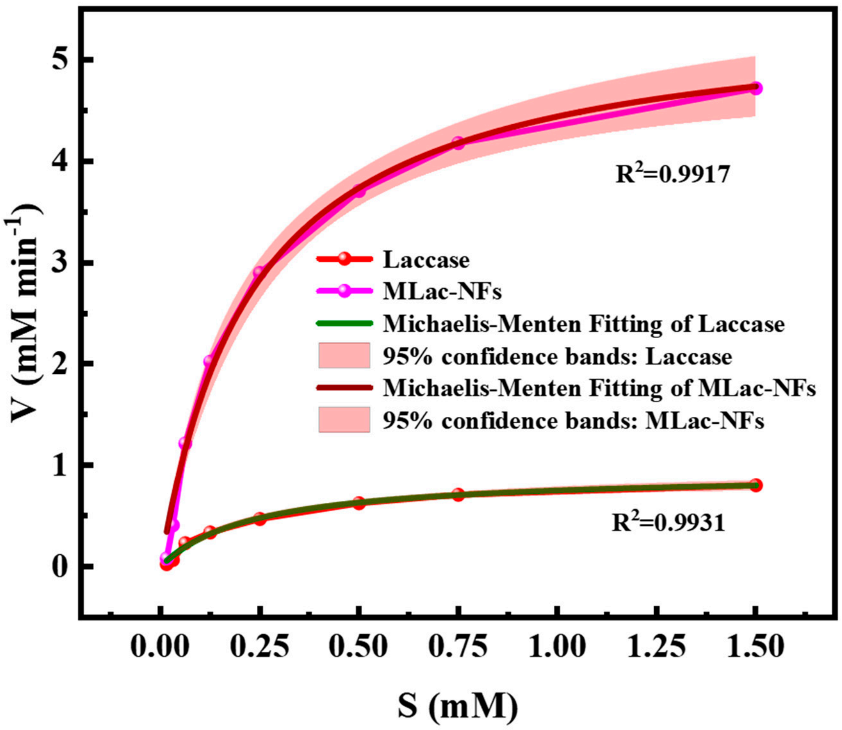

Figure S6. Enzymatic Kinetic.

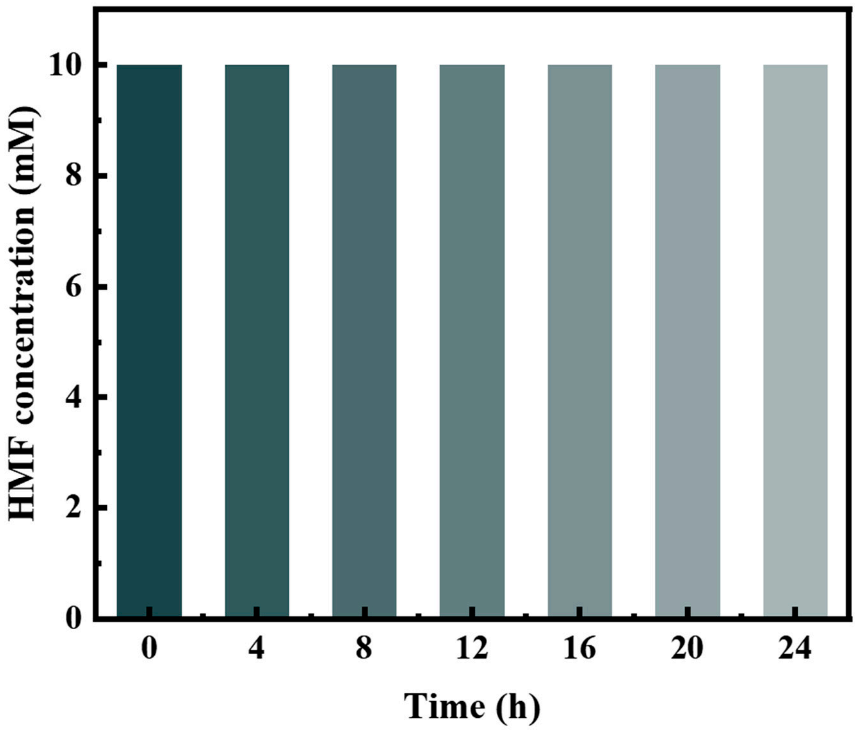

Figure S7. Magnetic material catalyzed oxidation of HMF.

Table S1. Retention times and detection wavelengths of each compound.

Compound	Retention times (min)	Wavelength (nm)
HMF	3.28	280
DFF	13.57	280
FFCA	9.83	280
FDCA	5.63	284
